# Critical Care Nurses’ Experiences Caring for Patients When Relatives Were not Allowed in the ICUs due to COVID-19 Pandemic

**DOI:** 10.1177/23779608221103627

**Published:** 2022-05-31

**Authors:** Lina Stenman, Lisa Högberg, Åsa Engström

**Affiliations:** 1 Critical Care Nurse, Skellefteå Hospital, Skellefteå, Sweden; 2 Critical Care Nurse, Lycksele Hospital, ICU, Lycksele, Sweden; 3 Department of Health, Education and Technology, Division of Nursing and Medical Technology, 91874 Lulea University of Technology, Luleå, Sweden

**Keywords:** visiting restriction, relatives, critical care nurse, family-focused care, intensive care unit, practice

## Abstract

**Introduction:**

Health care workers faced new challenges during the COVID-19 pandemic when physical contact with relatives more or less disappeared.

**Objectives:**

The aim of this study is to describe the experiences of critical care nurses (CCNs) working in intensive care units (ICUs) under the visiting restrictions imposed as a result of COVID-19.

**Method:**

This study followed a qualitative design. The purposive sample included CCNs with at least 1 year of experience working in an ICU with a visiting policy affected by the pandemic. Data collection was carried out via semi-structured interviews and analyzed through a qualitative content analysis with an inductive approach.

**Results:**

The study results are presented in three categories with 10 subcategories. CCNs value the presence of patients’ relatives at the bedside and described many challenges when relatives could not be present with the patient during the pandemic.

**Conclusion:**

Close relatives are able to share essential information about the patients and provide much-needed emotional support to them, the relatives’ role is of central importance and CCNs value their presence in ICUs more than any positive consequences of them not being there.

## Introduction

The COVID-19 pandemic challenged health care systems and societies worldwide, and millions of people became critically ill and required care in intensive care units (ICUs) (Murthy et al., 2020; Simpson & Robinson, 2020). On January 31, 2020, the first case of COVID-19 was confirmed in Sweden, and due to a rapid nationwide spread of the illness, the Swedish Parliament introduced a visiting restriction on the country’s special housing on March 30, 2020. Restricted visiting led to a temporary ban on outside visitors to increase patient and staff safety, and shortly thereafter, a restricted visiting ban was also introduced at many Swedish hospitals, wherein relatives were not allowed to visit during the patients’ care period, except for special cases, and nonurgent hospital visits were minimized. This study investigates the ways in which this restricted visiting ban affected critical care nurses (CCNs).

### Review of the Literature

Because the presence of relatives is an important component of caring for critically ill patients in an ICU, the impact of restricted visitation policies is a sensitive topic (Alexandersen et al., 2021; Andersson et al., 2021). ICU patients often experience difficulty communicating, and their relatives are able to provide invaluable information to the nursing staff; this information typically emerges after a long-term relationship between two individuals, such as between the patient and relatives (Engström & Söderberg, 2007b). In this context, these people may be a biological relative, partner, or even a close friend (Engström & Söderberg, 2007a).

Family-focused nursing is categorized as either family-related or family-centered nursing (Benzein et al., 2017; Nettina, 2014). Benzein et al. (2017) described family-centered care as a systematic approach wherein all members of the patient’s family are building blocks who support one another and asserted that this approach should have a more significant place in medical and nursing care; when considered together, the whole forms a slightly larger entity than when each building block is considered separately, and interactions between the patient and their relatives provide additional security (Benzein et al., 2017; Park et al., 2018). Hart et al. (2020) confirmed the increased need for family-centered nursing in difficult situations and emphasized the importance of finding alternative solutions to maintain family-centered nursing when physical visits were not possible.

Close relatives of a critically ill patient experience stress and feelings of fear, and these feelings escalate if they are not allowed to visit their seriously ill loved one (Valley et al., 2020). When given the opportunity to attend to the patient, they experience a greater sense of control (Davidson et al., 2017). Monroe and Wofford (2017) suggested that unrestricted visiting hours for relatives has the potential to motivate the patient to continue to fight and leads to increased satisfaction among relatives who are able to take part in the patient’s progress of care. Moreover, Goldfarb et al. (2017) reported that ICU patients with relatives who are physically present at the hospital require a shorter care period.

According to Akbari et al. (2020), while longer visits with close relatives stabilize a patient’s physiological parameters, short visits can be stressful for the patient. Previous studies (e.g., Martinez et al., 2012; Munshi et al., 2021; Rosa et al., 2017) noted that patients whose relatives are not allowed to be present demonstrate a greater tendency to develop delirium than patients whose relatives regularly visited them. Because it is essential to reduce the spread of infection during an ongoing pandemic, however, Munshi et al. (2021) highlighted the importance of limiting the number of relatives and insisted that all relatives test negative for COVID-19 before visiting, have good personal hygiene and wear appropriate face masks during visits. Because health care workers faced new challenges during the COVID-19 pandemic when physical contact with relatives more or less disappeared, the aim of this study is to describe the experience of CCNs who worked in ICUs under the restricted visiting ban.

## Method

### Design

Following a qualitative method, this descriptive qualitative study involved semi-structured interviews and content analysis (Polit & Beck, 2017). This study design was selected because interviews are the best method to gather descriptions and experiences, and the interviewers followed the consolidated criteria for reporting qualitative research (i.e., COREQ) (Flick, 2018; Tong et al., 2007).

### Participants Inclusion Criteria

The 10 study participants were selected through purposive sampling to ensure everyone had experienced the phenomenon being studied (Streubert & Carpenter, 2011). The study inclusion criteria were CCNs who had worked in an ICU for a minimum of 1 year, and who had worked closely with patients during the COVID-19 pandemic and experienced limited opportunities for relatives to physically attend the seriously ill ICU patient.

### Context

The nurses who participated in the study worked in five ICUs throughout northern Sweden, each of which had introduced a visitor visiting restriction due to the COVID-19 pandemic. Prior to the imposition of the visit ban, patients’ relatives were granted generous visiting hours and allowed to attend to their loved ones to a significant extent, and the CCNs actively involved them in the patients’ care. It should be noted that the visiting restriction did not apply to patients who were younger than 18 years of age or patients with disabilities who required assistance and exceptions were made in some situations, such as end-of-life care and severe life-threatening injuries or illnesses.

### Data Collection

CCNs who met the inclusion criteria were sent an invitation to participate in the study. Additional information about the study was provided to everyone who was interested in participating, and they were given the opportunity to ask questions. Every study participant provided written consent before their interview was conducted; prior to each interview, the participants were also given verbal informed consent information and instructed that they could withdraw from participating in the study at any time without needing to state their reason. The participants and the study authors jointly decided when their interviews would be conducted.

Using an interview guide (Table 1), the participants completed a one-on-one interview with the first or second author. The personal qualitative interviews were conducted throughout 2021 and were digitally audio-recorded. Due to the COVID-19 pandemic restrictions, the interviews, which lasted between 20 and 60 min, were conducted by telephone. Because all 10 interviews described similar experiences, a pattern was created that the authors felt served as an adequate basis for the results; this is also referred to as “saturation” (Polit & Beck, 2017).

**Table 1.** Interview Guide.

What was your visitor policy before the pandemic?
How did it change?
How did you involve your patients’ relatives before the pandemic? How did this change during the pandemic?
How did you experience these changes?
Were exceptions to the restraining order made? If so, how did you experience these exceptions?
Please describe how you handled contact with relatives during the pandemic? How did it feel?
If you used alternative communication solutions to involve relatives, can you describe them and how you experienced them?
How do you think the patients experienced the restraining orders?
How do you feel relatives were affected by not being able to have contact with their relatives?
What ethical challenges did you experience because of the restraining order? Do you think it affected your working group and working methods?
Did anything else about your work change because relatives were unable to visit?
Follow-up questions: Can you tell…? What did you think…? How did you feel…? Can you give examples…?

The interviews were digitally audio-recorded, then transcribed, after which the authors listened to the interviews and read through the transcripts to ensure that the material was consistent with the data collected. The collected data were then coded and de-identified to ensure a confidential presentation of the results.

### Data Analysis

The authors applied a qualitative content analysis with an inductive approach to the interview text, as described by Graneheim and Lundman (2004). The authors read the full text of the interviews and the meaning units that responded to the study aim, which were then extracted. These meaning units were cut down in such a way that they did not lose their meaning, known as condensation; they were then given a code that briefly described the core content thereof. Codes with similar content were sorted into subcategories, and the text in these subcategories was summarized into main categories. The three study authors had worked as CCNs; while this enabled them to ask the “right” questions, they were also aware of the risk of making judgements on the data based on their previous experience. For this reason, the authors systematically analyzed the data and discussed each step, independently verified the analysis, and discussed their findings before reaching a final agreement. The validity of this study has been ensured by reviewing the analysis in a seminar setting and by continual supervision by the third author who has wide experience in this kind of research.

### Ethical Considerations

The ethical committee in Sweden approved the study (Dnr 2020-02805). The heads of the ICU departments gave permission to conduct the study. Based on the information requirement, the participants were given information about the study, its background, and purpose and the manner in which it would be carried out. Written consent was obtained from all participants before the start of the study, and they were also given information about volunteering and their right to suspend participation at any time (CODEX, 2021). In accordance with current legislation and confidentiality requirements, study material and personal data about the participants were treated confidentially (CODEX, 2021). All data were stored on devices with code locks to ensure that only the authors had access.

## Results

A total of 10 nurses from five ICUs—two men and eight women—participated. The participants’ ages at the time of the interview ranged from 30 to 62 years, and their ICU work experience varied from 1 to 21 years of experience (*mean* = 9 years). Nine participants had a specialist education in intensive care and a master’s degree in nursing, and one participant was a trained ICU nurse. Everyone had worked in an ICU during the COVID-19 pandemic and observed the changed visitor policy in their unit.

The findings are presented in three categories—“The absence of relatives provides insight into their significance”; “Challenges when relatives are not allowed to be present”; and “A changed way of working”—with 10 subcategories. The categories and subcategories are presented in Table 2 and illustrated with specific quotations from the interviews in the text below.

**Table 2.** Overview of Findings.

Categories (*n* = 3)	Subcategories (*n* = 10)
Absence of relatives provides insight into their significance	The importance of patient’s close relatives The absence of relatives reduces the CCNs’ patient awareness CCNs’ experiences of how relatives are affected when unable to visit
Challenges when relatives are not allowed to attend	Difficulties caused by the restraining orders Difficulty conveying information Emotional difficulties with telephone Communication
A changed way of working	Altered communication The patient’s recovery opportunity Impact on the work environment Lessons learned from restraining orders

CCNs = critical care nurses.

### Absence of Relatives Provides Insight Into Their Significance

#### Importance of patient’s close relatives

According to the CCNs, it was more difficult to meet the patients’ needs for support, security and motivation when their relatives were not allowed to visit the ICU due to visiting restrictions. They explained that close relatives play an integral role in the well-being of ICU patients, and the security provided by relatives who are present can potentially reduce the risk of the patient suffering from such complications as confusion or depression.

You feel safe when relatives are there, sitting with them and holding their hand. (CCN 7)

The CCNs asserted that because it is not possible to know the significance of a patient’s relatives when they initially arrive in the ICU, they tried to enable patients to hear their relatives’ voices through telephone or video call, despite the visiting restrictions; this was even done when the patients were unable to communicate.

#### Absence of close relatives reduces CCNs’ patient awareness

The CCNs explained that reduced contact with relatives as a result of the visiting restrictions limited their knowledge of and weakened their relationship with the patient. They believed that the imposition of visiting restrictions underscored the importance of relatives providing information about the patient, such as details about each patient’s social background and their personal preferences; this was particularly obvious when the patient was unable to provide any information. Reduced patient awareness caused the CCNs to objectify their patients, and they admitted that it was easy to only focus on treating the patient’s disease when they did not have a holistic view of each patient.

The patients became de-identified in some way. It was like I took care of a—I don’t know what—like I only cared for one thing. (CCN 9)

Because the visiting restriction ban made physical visits impossible and most communication took place over the telephone, the CCNs’ relationship with their patients’ relatives became less personal. The CCNs felt that decreased personal contact with the patient made it easier for them to not bring their work-related thoughts and feelings home at the end of the day.

#### CCNs’ experiences of how relatives are affected when unable to visit

The CCNs feel that visits in the initial stages of the patient’s disease are more for relatives’ benefit than that of the patient; to make it easier for relatives who were not allowed to visit during this stage, the CCNs conducted video calls for patients and their relatives. They described the uncertainty experienced by relatives when they could not visit their critically ill relative, and they explained that everyone has a different reaction when someone they care about is critically ill, and this situation was even more difficult to deal with because of the visiting restrictions.

Everyone reacts differently when … their relatives become ill or are injured. Processing a traumatic event is not easy. (CCN 6)

According to the CCNs, most of the relatives understood why they were not allowed to visit. The CCNs felt that relatives who required more support and information were more significantly impacted by the visiting restrictions; the CCNs stated that despite daily telephone communication, being unable to physically visit with their loved ones caused many relatives to feel excluded, because when relatives are able to see the patient, it is easier for them to understand the course of the patient’s disease.

Some relatives, they just buy the location … well, okay we understand … and then there are those who feel that oh no … this is going to be really bad, and they probably all think so, but some express it so clearly. (CCN 7)

### Challenges When Relatives are not Allowed to be Present

#### Difficulties caused by visiting restrictions

Despite a clear visiting policy, the CCNs were uncertain when they could make exceptions to the visiting restrictions. They felt it was unfair if two patients had the same illness or injury, and the relatives of one of the patients were allowed to visit, while the other patient’s relatives could not. In some cases, the nurses stated that relatives and patients also interpreted this as abusive and reacted badly when other patients were allowed to have visitors.

Ethically, it was not easy for some to visit and some to not be allowed. It was tough to not to be able to have all the relatives here in the ICU. (CCN 6)

It was very difficult when the CCNs were required to refuse a relative’s visit because the patient’s condition had improved. Relatives had difficulty accepting that they were not allowed to come to the department and were limited to telephone contact. In the event that the patient’s condition deteriorated, the CCNs reported that decisions regarding visiting restrictions exemptions often came too late; because the guidelines were too strict, the patient’s and relatives’ final moments together were limited.

When the patient becomes so ill that you think they are going to pass away and you know that there is a visiting restrictions and it takes a long time to get a decision …we now call relatives here… Sometimes I think it’s been too long and if they had been told earlier, they would have come earlier, and they could have sat with their dying loved one a little longer. (CCN 4)

We had a dog here on one occasion. A patient was going to die, and he wanted to see his dog here at the hospital. It was really an exception. (CCN 2)

#### Difficulties conveying information

The CCNs explained that misunderstandings arose when close relatives did not have an opportunity to create their own image of the critically ill ICU patient. When a patient suddenly deteriorated and their level of care changed, the CCNs found it difficult to call and explain to the relatives in a way that they could understand and absorb the information. The CCNs described feeling as if they needed to be the eyes and ears of the relatives and convey everything they saw and heard about the patient.

For most people, they do not even know what it means to be at ICU. They have no idea … what it looks like here. (CCN 2)

The absence of close relatives placed higher demands on both the care staff and those relatives. The CCNs described incidents in which a patient had already been in ICU for a period of time before the CCNs became responsible for caring for them, and when the CCNs needed to have telephone conversations with the patients’ relatives, it was difficult to convey changes in the patient’s condition without knowing what the relatives understood about the course of the disease. It was difficult for the CCNs to find a balance between conveying some hope and not giving too much hope during these conversations; in the CCNs’ experience, it is easier for relatives to understand a patient’s condition and the direction in which it is heading if they are able to see how critically ill the patient is with their own eyes.

It’s difficult to know whether you’re giving] too much hope or too little hope. It’s easier when the relatives can see …for themselves if the patient is critically ill or doing better. (CCN 3)

#### Emotional difficulties with telephone-only communication

The CCNs admitted that their relationships with and knowledge about their patients’ relatives deteriorated as a result of the visiting restrictions and that it was difficult to know the best way to convey necessary information. One significant ethical challenge was being required to deliver bad news over the telephone. It was difficult for the CCNs to provide appropriate support and comfort during the telephone conversations, because they could neither see nor understand the relatives’ reactions, and it was stressful to hear the sadness of the person on the other end of the phone, especially if they were alone without someone to support them.

It is much more difficult to feel the atmosphere and feel how the relative understands and receives the information. (CCN 6)

Having to deny a relative to ability to come and sit with their sick loved one is very, very hard. (CCN 7)

### A Changed way of Working

#### Altered communication

According to the CCNs, the visiting restriction order altered their typical working methods. Contact with relatives has always been an important part of intensive care, and the CCNs were required to adjust the manner in which they maintained contact with and conveyed information to relatives during the visiting ban. Whereas physical meetings were the norm before the visiting ban was imposed, the CCNs’ interactions with relatives were primarily through telephone communication.

To avoid a large number of telephone calls from different relatives, the CCNs urged relatives to appoint one person in the patient’s family to maintain the contact with the hospital; this person could then share all necessary information with everyone else. During the visiting restrictions, digital communication tools were used as often as possible to mimic physical visits; specifically, relatives engaged in video calls to connect with the patient. The CCNs described different experiences regarding the use of video calls to communicate; some units used tablets for video communication with relatives, even if the patient was asleep or seriously ill, while other units did not to provide video communication for patients who could not provide consent. Notably, every CCN described positive experiences during video calls.

I had a patient who missed their family very much during Christmas so we called when their family were celebrating Christmas at home. The patient was immensely happy—it’s impossible to describe their joy. (CCN 4)

#### Patient’s recovery opportunity

The CCNs described that the ICU patients’ needed rest and recovery was easier to achieve during the visiting restriction ban; otherwise, visits from close relatives sometimes mean that the patient did not get enough peace and quiet.

The patients are so sick and tired when they [are] here. [In the past,] relatives were sometimes here a little too often. (CCN 1)

The CCNs explained, however, that there were certain times during the visiting restrictions when the patients were less rested when their relatives were unable to visit because the care staff was nursing, conducting examinations, and sampling the entire time. The CCNs further stated that when they were constantly working with the patients, there was the risk that they would not get sufficient rest.

When relatives do not come to visit, you’re more likely to work with the patients all the time. (CCN 6)

#### Impact on work environment

As the CCNs pointed out, the presence of relatives during visits can sometimes be very time-consuming, and fewer visits due to the visiting restrictions therefore contributed to a calmer work environment; they also asserted that the climate in their working group was easier when relatives were not visiting, because they could laugh and have fun with non-work-related matters without worrying that the relatives would find their behavior insulting. Furthermore, during the visiting restrictions, the CCNs stated that they were better able to supervise all the patients for whom they cared.

When you are in the ward, you have more control over patients other than your own. … Because there are fewer people in the ward. … it is easier to keep track of all the patients. (CCN 6)

According to the CCNs, when they did not need to accommodate relative visits, they were free to plan and direct nursing efforts and examinations during their work shifts as they wished. For example, prior to the imposition of visiting restrictions, patient mobilization sometimes needed to be postponed if relatives were visiting, because the CCNs believed it was more important for the patients and their relatives to spend time together.

We have been able to plan our entire session. … How we work … wash, bed and take care of the patient. We have never had to worry about relatives arriving. (CCN 7)

#### Lessons from new working conditions

The CCNs described several lessons they learned while working with restricted visiting arrangements. Because ICU patients are critically ill, it is difficult to limit their visiting hours to the same extent as other care units. The CCNs further stated that improvements in the patients’ conditions related to optimized patient rest led them to believe that it might be advisable to consider fixed visiting hours when the visiting restriction is lifted. Finally, the CCNs said they plan to continue stressing the importance of good personal hygiene when close relatives visit patients to reduce the risk of spreading infection into or out of the hospital after the visiting restriction was lifted.

Maybe we will be a little more careful now …because we see the stomach ailments—and yes, we escaped a lot of illness thanks to people using hand sanitizer and wearing facemasks. (CCN 8)

Video conversations with relatives were described as a means of communication that could continue to be used when the order restricting visits was lifted. When patients’ relatives live far away and do not have the opportunity to make physical visits, the CCNs asserted that video calls could help promote and maintain contact between relatives and patients and help the CCNs to create a deeper understanding of the patient’s condition to their relatives and explain what intensive care means.

I think video calls were great, and they are definitely something we could use a lot more. Relatives who live far away could also be communicated with in other ways. (CCN 4)

Finally, the CCNs expressed that a significant part of ICU care entails caring for and meeting with their patients’ relatives, and the nurses missed having them present in the unit.

The patients did not have relatives present, and I missed them. … There are sometimes too many relatives in the ICU, but it’s absolutely not good to be without them. (CCN 10)

## Discussion

CCNs in ICUs see tremendous value in visits from their patients’ relatives, which not only benefit the patients and their relatives, but also the CCNs. This can be interpreted according to Swanson’s (1993) nursing theory, a model that was created to guide nurses on the proper way to meet with patients and relatives. According to Swanson, nursing consists of five steps or processes—“maintaining belief”, “knowing”, “being with”, “enabling”, and “doing for”—which describe a nursing approach that is capable of creating a structured work environment and facilitating communication between a nurse, their patients and their patients’ relatives to achieve well-being and health.

According to the CCNs, prior to the visiting restriction, they enlisted the help of relatives to strengthen their patient’s hope and belief in the future, and it became more challenging to maintain the patient’s fighting spirit when their relatives were not allowed to visit physically. The CCNs’ actions to attain help from the patients’ relatives can also be explained by Swanson’s (1993) concept of “maintaining belief”, in which the aim of a nurse’s work should be to strengthen the patient’s sense of hope, faith in the future and meaningfulness. Our results underscored the CCNs’ belief that patients and their relatives are all strengthened when relatives are allowed to physically visit with the patient. This has been confirmed in previous studies, which found that the presence of relatives tends to be a positive step in the patient’s recovery (Alexandersen et al., 2021).

Our study also showed that the CCNs feared giving their patients’ relatives too much or too little hope. This finding is also in line with Swanson’s (1993) concept of “maintaining belief” when nurses meet with relatives to maintain the relatives’ hope and belief in the abilities of others. Previous studies have described many emotions experienced by relatives of ICU patients—such as anxiety, fear, insecurity, and varying degrees of hope—and they also emphasized that nurses can help those who are close to patients by acknowledging their feelings and supporting them throughout the care period (Valle & Lohne, 2021; Vasconcelos et al., 2016).

According to the CCNs, while some relatives expressed a tremendous need to visit the patient and see for themselves how their loved one was doing, others wanted less contact with health care. It was up to CCNs to identify and respond to the relatives based on their expressed needs and preferred level of engagement (Fergé et al., 2021). We found that exceptions to the visiting restriction were made to meet the needs of close relatives at times. Being close to a patient in the ICU often involves a great deal of mental strain and is associated with significant anxiety (Valley et al., 2020). Previous studies (e.g., Andresen et al., 2015; Fergé et al., 2021) described an increased risk that relatives of patients in ICUs may experience post-traumatic stress disorder (PTSD) due to the psychological strain it entails and observed the importance of flexible visiting hours to identify those at risk of developing PTSD to provide support, assistance, and all necessary information. Moreover, according to the CCNs in the present study, despite attempts to involve their patients’ relatives as a natural part of care, visiting restrictions significantly hampered these efforts; this concurs with Wetzig and Mitchell (2017), who concluded that relatives only feel truly involved in nursing when they are allowed to visit with their loved one.

In this study, the CCNs stated unequivocally that relatives’ information about their patient improved the level of care they could provide to their patients. Swanson (1993) described the concept of “knowing” as striving to understand every patient’s personal situation to adapt medical care to each individual patient. According to Cederwall et al. (2018), because ICU patients are often seriously ill and sedated, it can be difficult to provide person-centered care in this setting. The CCNs in the present study also pointed out that personal information is especially vital when their patients are unable to speak; the necessity of involving relatives when a patient is incapable of communicating was also confirmed in earlier research (e.g., da Silva & Henricson, 2013; Engström et al., 2011).

When relatives were barred from visiting and prevented from providing knowledge about the patients, the CCNs reported the experience of objectifying the patients. Andersson et al. (2021) described this same phenomenon wherein it was difficult for CCNs to see their patients as people during the COVID-19 pandemic, because they did not know anything about their interests, needs or resources because of a lack of contact with their patients’ relatives during the visitation ban. Our results further highlighted nurses’ challenges to maintain an emotional presence to the same extent as before the restrictions, and the CCNs explained the ways in which minimal knowledge about their patients made it more difficult to understand the situations of both the patients and their relatives. In this context, the nurses’ emotional presence for their patients is in line with Swanson’s (1993) concept of “being with”.

According to the CCNs in this study, contact with patients’ close relatives served as a link to the outside world and allowed the patient to know what they were coming home to after their care was concluded; involving their relatives is especially important in situations where there is physical distancing, such as providing assistive technological solutions (Hart et al., 2020). During the visiting ban, the CCNs worked with other professions in a solution-focused manner to identify alternative ways to facilitate their patients’ transitions out of the ICU and changes that would be entailed in this process. This can be explained by Swanson’s (1993) concept of “enabling”, which requires that nurses strengthen their patients and teach them new approaches to contend with life changes.

In this study, video communication was employed as an alternative solution for maintaining contact with relatives. Because video calls provide visual and auditory cues to patients and their relatives, they provide an experience that is more similar to physical visits than telephone calls alone (Fang et al., 2020; Schnock et al., 2017). The CCNs in this study reported that it was difficult to provide appropriate emotional support and comfort via telephone calls. This is supported by Kennedy et al. (2021), who described relatives’ positive experiences when given the opportunity to see the patient and their surroundings; other studies (e.g., Freeman-Sanderson et al., 2020; Negro et al., 2020) found that connecting patients and their relatives through both audio and visual video calls enhanced the relatives’ understanding of their loved one’s medical situation. Notably, our findings also indicated that there were ethical challenges related to knowing when to use video communication, such as when a patient was unable to consent; this is in line with limitations and challenges related to virtual visits and video communication to offer relatives insights into the medical situations of anesthetized patients while continuing to protect the patient’s integrity that was previously described by Rose et al. (2020).

The CCNs in this study admitted that it was difficult to clearly explain what intensive care was and describe what was being done to help the patient when they were only able to speak to relatives over the telephone. This can be linked to previous research evidence (e.g., Piscitello et al., 2021; Wetzig & Mitchell, 2017), which emphasized the importance of helping relatives to properly understand their loved one’s medical situation by receiving information that is adapted to their personal levels of understanding and having the opportunity to visit and speak to medical professionals in person. In the present study, CCNs explained that when close relatives were able to be physically present, they could see what the CCNs were describing and were thus able to more easily understand what the nurse was attempting to convey. This was confirmed by Kennedy et al. (2021), who described the need expressed by close relatives to see their loved ones via video to better understand the situation better when they were prevented from visiting.

Interestingly, the CCNs in this study reported that the visit restrictions actually facilitated their medical care by allowing them to plan their work without the distraction of having relatives present. Despite these favorable factors, however, our results overwhelmingly showed how important ICU patients and their relatives are to one another and demonstrated the CCNs’ willingness to look past their own work situation to do what was in the patient’s best interest. In his theory, Swanson (1993) described the concept of “doing for” as nurses performing medical care with a holistic view of their patients and a desire to care for them, in the same manner, they would want to be treated if they were the patient. The CCNs in this study insisted that they were prepared to make any necessary sacrifices in their work environment to enable interactions between their patients and their patients’ relatives; this sincere willingness could be due to the CCNs envisioning what they would want their own relatives to experience if they were faced with a serious illness.

When asked to describe lessons they learned as a result of the visiting restrictions, the CCNs reflected on their positive experiences with video communication and asserted that this mode of communication should continue to be used to a significant extent after the visiting ban is lifted; several years before this study, Brecher (2013) investigated the use of video communication and found that this technology had the potential to enhance and improve contact between patients and their relatives. The CCN study participants also pointed out that, despite living in a highly technological society, video communication is insufficiently used in the medical context; from a societal perspective, this is an important lesson for a means of optimizing medical care and increasing the well-being of patients and their relatives, and by extension, the work satisfaction of CCNs.

### Strengths and Limitations

While the participants, setting, data collection method, and analysis of this study were thoroughly described to allow readers to evaluate the findings’ transferability to other contexts and the participants’ confidentiality was ensured, this study is limited because it only explores the experiences of CCNs working in Swedish ICUs. Furthermore, due to the ongoing COVID-19 pandemic and in accordance with the regulations of the Swedish Public Health Agency’s regulations and general advice on responsibly preventing the spread of the COVID-19 virus, the interviews were conducted by telephone; even though the participants were also given the opportunity to conduct their interviews via video, everyone chose to conduct their interviews over the telephone. Brinkmann and Kvale (2018) highlighted the importance of paying attention to the participant’s tone of voice when body language and eye contact were not possible due to interviews being conducted via telephone, and they described the necessity of responsive interviewers asking the right follow-up questions at the right time to allow participants to think about their answers; but not being able to see the participant’s facial expressions and physical gestures made this very difficult. The interviews were rich in content, however, and there were several possible benefits to the telephone interviews, such as the participants feeling more at ease when talking to someone whom they could not see.

### Implications for Practice

One area of improvement that has emerged during the COVID-19 pandemic is the increasing use of alternative means of communication such as video calls to ensure consistent contact with close relatives who are unable to be present for various reasons. While future studies in this area are still needed, the results of this study demonstrate that the implementation of new approaches to facilitate visits from the relatives of ICU patients are able to promote the health and well-being of both the patients and their relatives while continuing to safeguard the relationship between the patient, their relatives, and CCNs.

## Conclusion

Because close relatives of ICU patients are able to share essential information with CCNs and provide much-needed emotional support to the patient, their role is of central importance and CCNs value their presence in ICUs more than any positive consequences of them not being there. This strengthens previous evidence that family-centered care should be prioritized in ICUs to enhance the well-being of patients, their relatives, and staff. In the event of limited or restricted visitation, such as those imposed by the visiting restriction during the COVID-19 pandemic, technical solutions and resources are essential so patients, their relatives, and staff can continue to maintain close contact. There remains a lack of research on the experiences of patients and their relatives when their ability to visit is inhibited, and this should be investigated in future research.
